# Supplementary material for: Human motor fatigability as evoked by repetitive movements results from a gradual breakdown of surround inhibition
Source: eLife. 2019 Sep 16;8:e46750. doi: 10.7554/eLife.46750 (PMC6746551; doi:10.7554/eLife.46750)
Supplement: Transparent reporting form [file elife-46750-transrepform.pdf]

eLIFE
1st Floor
24 Hills Road
Cambridge CB2 1JP, UK
P 01223 855340
W elifesciences.org
T @elife

eLife's transparent reporting form

We encourage authors to provide detailed information *within their submission* to facilitate the interpretation and replication of experiments. Authors can upload supporting documentation to indicate the use of appropriate reporting guidelines for health-related research (see EQUATOR Network), life science research (see the BioSharing Information Resource), or the ARRIVE guidelines for reporting work involving animal research. Where applicable, authors should refer to any relevant reporting standards documents in this form.

If you have any questions, please consult our Journal Policies and/or contact us: editorial@elifesciences.org.

Sample-size estimation

- You should state whether an appropriate sample size was computed when the study was being designed
- You should state the statistical method of sample size computation and any required assumptions
- If no explicit power analysis was used, you should describe how you decided what sample (replicate) size (number) to use

Please outline where this information can be found within the submission (e.g., sections or figure legends), or explain why this information doesn't apply to your submission:

eLife Sciences Publications, Ltd is a limited liability non-profit non-stock corporation incorporated in the State of Delaware, USA, with company number 5030732, and is registered in the UK with company number FC030576 and branch number BR015634 at the address 1st Floor, 24 Hills Road, Cambridge CB2 1JP | August 2014

We calculated the sample sizes for the behavioral experiments from our previous work reported in (1-4). From these studies we had a sample of 127 participants who all acquired a 4 element finger sequence task over 12 trials, each lasting 30s. Even though these data are quantified by a performance index, this metric does mainly effect tapping speed. Using this parameter we quantified a proxi of motor slowing for the last of these trials where sequence learning has reached a plateau. Analyzing the difference in between the first and the last 10s bin of sequence tapping revealed a clear effect and a Cohen's d effect size of 0.92. Based on this value, we estimated a required sample size of n=12 for a dependent, two-sided t-test with a power of 0.8 and a=0.05 using G Power. This data is available as a xls spreadsheet.

We are not aware of prior data that would have allowed us to estimate which sample size was required for the fMRI, TMS or EEG experiments. Therefore, we applied conventional estimates, i.e. 25 participants for the fMRI experiment (5) and at least 12 participants for the TMS/EEG experiments.

(1) Fattinger, S., de Beukelaar, T. T., Ruddy, K. L., Volk, C., Heyse, N. C., Herbst, J. A., ... & Huber, R. (2017). Deep sleep maintains learning efficiency of the human brain. *Nature communications*, *8*, 15405.

(2) De Beukelaar, T. T., Alaerts, K., Swinnen, S. P., & Wenderoth, N. (2016). Motor facilitation during action observation: The role of M1 and PMv in grasp predictions. *Cortex*, *75*, 180-192.

(3) De Beukelaar, T. T., Van Soom, J., Huber, R., & Wenderoth, N. (2016). A day awake attenuates motor learning-induced increases in corticomotor excitability. *Frontiers in human neuroscience*, *10*, 138.

(4) De Beukelaar, T. T., Woolley, D. G., & Wenderoth, N. (2014). Gone for 60 seconds: reactivation length determines motor memory degradation during reconsolidation. *Cortex*, *59*, 138-145.

(5) Durnez, J., Degryse, J., Moerkerke, B., Seurinck, R., Sochat, V., Poldrack, R., & Nichols, T. (2016). Power and sample size calculations for fMRI studies based on the prevalence of active peaks. *BioRxiv*, 049429.

Replicates

- You should report how often each experiment was performed
- You should include a definition of biological versus technical replication

eLife Sciences Publications, Ltd is a limited liability non-profit non-stock corporation incorporated in the State of Delaware, USA, with company number 5030732, and is registered in the UK with company number FC030576 and branch number BR015634 at the address 1st Floor, 24 Hills Road, Cambridge CB2 1JP | August 2014

- The data obtained should be provided and sufficient information should be provided to indicate the number of independent biological and/or technical replicates
- If you encountered any outliers, you should describe how these were handled
- Criteria for exclusion/inclusion of data should be clearly stated
- High-throughput sequence data should be uploaded before submission, with a private link for reviewers provided (these are available from both GEO and ArrayExpress)

Please outline where this information can be found within the submission (e.g., sections or figure legends), or explain why this information doesn't apply to your submission:

We did not include technical or biological replications in a strict sense. However, throughout our series of experiments we included many conceptual replications and repeatedly demonstrated the behavioral effect. In total 122 participants were tested including 12-25 participants per experiment and we consistently observed large effect sizes for motor slowing (all cohen's d > 1.04).

Statistical reporting

- Statistical analysis methods should be described and justified
- Raw data should be presented in figures whenever informative to do so (typically when N per group is less than 10)
- For each experiment, you should identify the statistical tests used, exact values of N, definitions of center, methods of multiple test correction, and dispersion and precision measures (e.g., mean, median, SD, SEM, confidence intervals; and, for the major substantive results, a measure of effect size (e.g., Pearson's r, Cohen's d)
- Report exact p-values wherever possible alongside the summary statistics and 95% confidence intervals. These should be reported for all key questions and not only when the p-value is less than 0.05.

Please outline where this information can be found within the submission (e.g., sections or figure legends), or explain why this information doesn't apply to your submission:

All information can be found in the methods section: Description of statistical analysis including used tests and methods of multiple test correction can be found under data analysis of the respective experiments. N is described under the subsection participants and indicated in the figure legends. Effect sizes, summary statistics and exact p-values are presented in the results section. All summary data are depicted as mean +/- SEM and are indicated as such, if not stated otherwise. We also uploaded a spread-sheet summarizing the statistical tests performed in each experiment.

(For large datasets, or papers with a very large number of statistical tests, you may upload a single table file with tests, Ns, etc., with reference to sections in the manuscript.)

Group allocation

- Indicate how samples were allocated into experimental groups (in the case of clinical studies, please specify allocation to treatment method); if randomization was used, please also state if restricted randomization was applied
- Indicate if masking was used during group allocation, data collection and/or data analysis

Please outline where this information can be found within the submission (e.g., sections or figure legends), or explain why this information doesn't apply to your submission:

All experiments in this study are within-subject designs. Therefore, there was no group allocation necessary. In experiment 3, 6 and 7 participants performed control and motor slowing tapping in two different sessions, this is stated in the methods section and session order (i.e. if control or motor slowing tapping was performed in the first session) was counter-balanced across participants.

Additional data files ("source data")

- We encourage you to upload relevant additional data files, such as numerical data that are represented as a graph in a figure, or as a summary table
- Where provided, these should be in the most useful format, and they can be uploaded as "Source data" files linked to a main figure or table
- Include model definition files including the full list of parameters used
- Include code used for data analysis (e.g., R, MatLab)
- Avoid stating that data files are "available upon request"

Please indicate the figures or tables for which source data files have been provided:

Source data for the most important bar plots depicted in the figures is available as spread sheet.

eLife Sciences Publications, Ltd is a limited liability non-profit non-stock corporation incorporated in the State of Delaware, USA, with company number 5030732, and is registered in the UK with company number FC030576 and branch number BR015634 at the address 1st Floor, 24 Hills Road, Cambridge CB2 1JP | August 2014
